# Forward dynamic optimization of handle path and muscle activity for handle based isokinetic wheelchair propulsion: A simulation study

**DOI:** 10.1080/10255842.2018.1527321

**Published:** 2018-11-06

**Authors:** Nithin Babu Rajendra Kurup, Markus Puchinger, Margit Gföhler

**Affiliations:** Research Division for Biomechanics and Rehabilitation Engineering, TU Wien, Vienna, Austria

**Keywords:** Wheelchair propulsion, musculoskeletal modelling, handle-based propulsion, dynamic optimization

## Abstract

Push-rim wheelchair propulsion is biomechanically inefficient and physiologically stressful to the musculoskeletal structure of human body. This study focuses to obtain a new, optimized propulsion shape for wheelchair users, which is within the ergonomic ranges of joint motion, thus reducing the probability of injuries. To identify the propulsion movement, forward dynamic optimization was performed on a 3D human musculoskeletal model linked to a handle based propulsion mechanism, having shape and muscle excitations as optimization variables. The optimization resulted in a handle path shape with a circularity ratio of 0.95, and produced a net propulsion power of 34.7 watts for an isokinetic propulsion cycle at 50 rpm. Compared to push-rim propulsion, the compact design of the new propulsion mechanism along with the ergonomically optimized propulsion shape may help to reduce the risk of injuries and thus improve the quality of life for wheelchair users.

## 1. Introduction

Significant research work has been performed over the decades, to understand the biomechanical and physiological factors involved in wheelchair propulsion (van der Woude,Veeger et al. 2001), as wheelchairs are considered an important necessity for the daily mobility and ambulation for physically disabled and injured persons.

van der Woude, Dallmeijer et al. (2001) reported that the hand-rim was the most favoured mode of propulsion by a large percentage of wheelchair users even though it follows the least efficient pattern of propulsion. The use of the hand-rim may lead to severe upper limb injuries mainly in the shoulder joint such as rotator cuff tear and injuries in the wrist region caused by the discontinuous and complex upper limb movements during propulsion (Arnet et al. 2012). Studies focused on the kinematic aspects of push rim propulsion, have shown that the joints of the upper limb exhibit large ranges of motion and at certain extreme joint limits, the muscles may need to produce relatively large forces to maintain the propulsion cycle. In such situations the muscles operate in unfavourable regions of their force–length curves, resulting in limited force production and subsequently leading to musculoskeletal injury and pain (Rao et al. 1996; Wei et al. 2003).

In addition, studies on the kinematics and kinetics of wheelchair propulsion have reported that increasing the velocity of propulsion leads to increase in shoulder forces and moments (Mercer et al. 2006; Gil-Agudo et al. 2014). The increased magnitude of reaction forces at high speeds due to low contact duration (Desroches et al. 2010; Russell et al. 2015) can impose high mechanical demand on the shoulder muscles which control stabilization and rotation and this may contribute to acute shoulder pain and injury. Boninger et al. (2002) noted that stroke patterns at decreased cadence resulted in lower cases of medial nerve injuries due to longer contact duration with the push-rim. Jayaraman et al. (2015) had reported that push-rim propulsion can lead to higher jerk forces due to sharp direction changes and abrupt speed changes associated with propulsion. Hence restricting the joint motion to ergonomic limits as in this study can prevent injuries due to simultaneous occurence of peak forces and peak shoulder angles with increasing speed as observed in push-rim propulsion (Koontz et al. 2002).

Arm-cranking and hub-crank wheelchairs are the only available devices that use a continuous cyclic motion for wheelchair propulsion. These devices have geometrical restrictions (e.g. large frame size) that make them unacceptable for use in daily living as they severely restrict the maneuverability in small spaces (Smith et al. 1983; Mukherjee and Samanta 2001). But cyclic form of propulsion is quite efficient as the force is uniformly applied to the handle over the full rotation resulting in lower peak force (Arnet et al. 2013). Whereas under hand-rim propulsion, additional braking moments are produced during the hand-rim contact and release periods, which hinder the forward propulsion movements (Kwarciak et al. 2009). These forces reduce propulsion efficiency and increase the loading on the joints (Arnet, Drongelen, et al. 2012; Arnet et al. 2013). In addition, the continuous circular propulsion helps to distribute the propulsion load over more muscle groups, mainly by involving the flexor and extensor muscle groups, thereby reducing the chances of overuse injuries of specific muscles (van der Woude,Veeger et al. 2001). Based on the above concepts there is a significant shortage of propulsion techniques, which incorporate the cyclic propulsion pattern of hand cycling while compact enough to be adapted to a conventional wheelchair for daily living.

Several studies have utilized three-dimensional upper extremity models and optimization techniques to estimate the muscle forces and joint variables involved in hand-rim wheelchair propulsion (Arnet, Drongelen, et al. 2012; Arnet, van Drongelen, et al. 2012; Morrow et al. 2014). Forward dynamic simulations have been widely used even though computationally expensive to understand the intermuscular coordination during hand-rim based wheelchair propulsion (Rankin et al. 2012; Rankin and Neptune 2012; Slowik et al. 2016).

To the best of our knowledge, no studies have targeted path shape optimization for wheelchair propulsion so far. Few studies dealt with shape optimizations using 3D human models and its concerned variables for dynamic chain-ring optimizations for cycle pedalling, a very similar problem for the lower instead of the upper extremity. Kautz and Hull (1995) performed forward dynamic optimization using a torque driven 3D model to identify an optimal non-circular chain ring shape for pedalling, but the study lacked the important intrinsic properties of muscles such as muscle length and shortening velocity which have an influence on the resultant optimal chain ring shapes. A subsequent study by Rankin and Neptune (2008) included a complete musculoskeletal model of the lower limb attached to a pedal setup for identifying chain ring shape using dynamic optimization. The results indicate that the muscle activation–deactivation dynamics play a vital role in determining the optimized chain ring shape.

The aim of this study is to establish a musculoskeletal model of the upper extremity and determine a handle-based continuous wheelchair propulsion movement in a forward dynamic optimization approach that optimizes the handle-path shape and muscle activity patterns for maximum net propulsion power.

## 2. Methods

### 2.1 Musculoskeletal model

**Figure 1.**
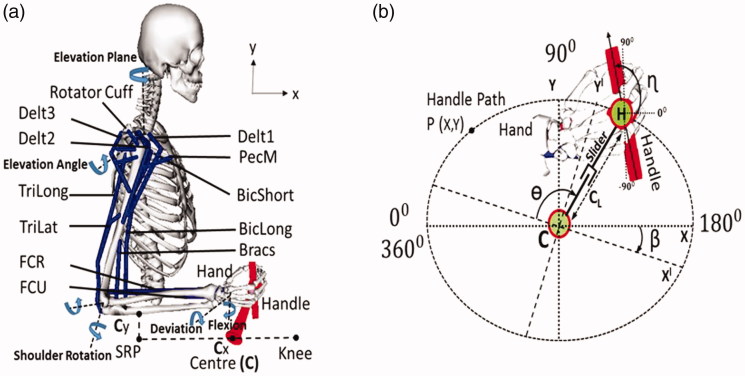
(a) Musculoskeletal model with right hand linked to the propulsion mechanism, with the 15 muscle actuators, Delt1(AnteriorDeltoid), Delt2(MiddleDeltoid), Delt3(PosteriorDeltoid), BicLong(BicepsLong), BicShort(BicepsShort), TriLong(TricepsLong), TriLat(TricepsLateral), Bracs(Brachialis), FCR(FlexorCarpiRadialis),FCU(FlexorCarpiUlnaris),PecM(Pectoralis Major) and Rotator cuff muscles(Supraspinatus (SUPRA), subscapularis (SUBSC), infraspinatus (INFRA), teresminor (TMIN)) with the major DOF such as Elevation Plane, Elevation angle, Elbow flexion, Shoulder rotation, Wrist deviation and flexion. (b) Kinematic components of the propulsion mechanism, with major DOF such as crank angle (ɵ), effective crank length (C_L_), tilt angle (β) and handle angle (ɳ).

The dynamic musculoskeletal model was developed in the OpenSim (Delp et al. 2007) platform, involving the anthropometry and muscle force-generating properties of a 50th percentile adult male based on the work by Saul et al. (2015). The rigid segments of the model included the fixed thorax segment (no spine movement), the right upper arm, the right forearm defined by individual components of ulna and radius, and the hand segment. The model was not bilaterally symmetric and only included the right shoulder and hand segments. The shoulder was modelled as a 3 DOF (Degree of freedom) joint comprising of the elevation plane, the shoulder elevation angle (thoracohumeral angle) and the shoulder rotation angle. The elbow joint is defined by 1 DOF with 0° (extension) to 130° (flexion). The wrist joint is modelled with 2 DOF, wrist flexion and wrist deviation (Holzbaur et al. 2005). The hand supination had to be constrained to restrict the motion of the hand in the plane during the path optimization. The collective motion of the shoulder girdle (humerus, clavicle and scapula) determines the motion of the shoulder joint. Humerus and scapula are articulated by a ball and socket joint, while regressive equations determine the motion of the shoulder girdle, which moves only with the elevation angle. The model included 15 musculotendon actuators, spanning the shoulder, elbow and the wrist joints as shown in Figure 1. A Hill type muscle model, defined by Thelen (2003) was used in this study, including both active and passive muscle force generation characteristics based on the muscle force–velocity and force–length relationships. The lumped muscle model included the 4 parameters (optimal fibre length, maximum isometric force, tendon slack length and pennation angle) used to represent the generic properties of musculotendon units (Arnold and Delp 2011). Elastic and damping joint torques were applied to the model to enforce the joint limits (Rankin et al. 2010). The novel handle based propulsion (HBP) mechanism is located in the parasagittal plane that contains the shoulder joint, with the crank centre coordinate C_XY_ fixed in the global frame (global frame origin at the sternum of the upper extremity model). Cx is the mid-point between the seat reference point (SRP) of the wheelchair and knee joint position of the model, considering the model is in a seated position on the wheelchair. C_Y_ is the vertical height from the SRP to the forearm of the model with elbow joint being flexed at 90°.

The propulsion mechanism consists of the crank that rotates around the origin C and a sliding segment which moves with respect to the crank and can change the effective crank length (C_L_) during rotation. The handle is linked to the sliding segment by a pin joint (H). The propulsion mechanism has 4 variables, the crank angle (ɵ), the effective crank length (C_L_), the tilt angle (β) and handle angle (ɳ). During propulsion the movement is defined by 2 DOF (crank angle and handle angle). The crank rotates in clockwise direction as depicted in Figure 1b. The crank’s effective length can have values between 0.030 m and 0.155 m. To connect the arm and the propulsion system, the hand and the handle segments are rigidly welded.

### 2.2 Optimization

Dynamic optimization and forward dynamic simulation were performed using OpenSim 3.2. For the optimization the Interior point optimization algorithm(IPOPT) on an Intel ®Xeon® CPUE5-1650 with 6 cores and clock speed of 3.50 GHz, on a 64-bit operating system was used. The dynamic optimization routine followed is a “fully forward” approach (Sharif Shourijeh and McPhee 2014). The neural muscle excitations u(t), and the shape parameters of the path (A, B, n, and β) (Equation 1 and 2) act as the control signals. The optimal solutions of these controls are found using dynamic optimization, in combination with solving the muscle dynamics and multibody system dynamics by integration at each iteration. A variable step size Runge–Kutta–Merson integrator (Hairer et al. 2008) was used in this study.

#### 2.2.1 Optimization criterion

Instantaneous power is obtained as the product of instantaneous torque around the crank times the crank speed at each point of the optimization, the average over one propulsion cycle gives the net propulsion power (Watts). The cost function is designed to maximize the net propulsion power over one complete propulsion cycle at each iteration, with added penalties to limit the joint motion within the physiological human limits as defined in the model and to the parasagittal plane defined by the wheels.

#### 2.2.2 Optimization parameters

For muscle excitation optimization, 10 control points at equal time intervals over a full crank rotation i.e., between the initial time (t_0_) and the final time (t_f_) were selected for each muscle. At each control point the neural excitation u(t), was optimized with values ranging between 0 for least excited muscle state to 1 for maximum excited state. A cubic spline function was used to interpolate the control nodes as cubic functions reduced the oscillations between the data points, and produce smoother interpolated data set when compared to other polynomial interpolators.

For handle shape optimization, the path of the handle was parameterized as a function of the crank angle (ɵ) as represented in the parametric Equation (1 and 2). The equations helps to prevent concave regions in the path, and also facilitates the generation of varied shapes for optimization (Von Seggern 2016).

(1) $$P_{X}(\theta )=A\;\text{cos}(\theta )$$

$$P_{Y}(\theta )=B\;\text{sin}(\theta ){\;\text{sin}}^{n}(0.5\theta )$$

(2) $$P{(}_{X}{{}^{l}}_{Y}{}^{l})=R_{z}\left(\beta \right)P_{\text{XY}}(\theta )$$

Four optimization variables (A, B, n, and β) define the shape of the path. The scaling factors denoted by A and B were constrained by the limits of the crank effective length C_L_. The shape factor n can have values ranging from 0 to 1. The final variable β (Equation 2) defines the tilt angle of the path P_XY_(ɵ) in clockwise direction with respect to the origin C. *R_z_* indicates the rotation matrix to rotate the path in the x–y plane. In total 154 optimization variables were used: 150 variables for muscle excitation, 4 variables from the parametric Equations (1 and 2). P_X_ and P_Y_ represent the x and y Cartesian coordinates of the shape (P_XY_).

During each forward simulation the motion of the HBP was realized by converting the Cartesian coordinates of the optimized path P_X_^l^_Y_^l^(ɵ), (Equation 2) to polar form and then prescribing the effective crank length (C_L_) as a function of crank angle (ɵ). In OpenSim, the prescribed motion of the slider joint is generated by inputting a linearly interpolated function of above parameters at each iteration of the optimization.

### 2.3 Test setup

The initial variables for the optimization were randomly generated for both shape and muscle excitation parameters, and were optimized to maximize the cost function for each crank cycle. For the muscle parameters, the initial excitation (control signal) and the activation values at time (t_0_) were set as 0.050, and 3 complete cycles were simulated to reach a steady state. After the third cycle, a constraint was enabled to set the muscle excitation values at time (t_0_) equal to the excitation values at time (t_f_), thereby creating a periodic muscle activity pattern for hand propulsion. In addition, a terminal constraint was applied such that at time (t_f_) the crank angle is 360°. In this study the angular velocity (ω) of the crank was set as constant (50 rpm) to emulate an isokinetic ergometer. The set constant 50 rpm speed, is the value required for over ground propulsion for daily living (van der Woude, Veeger et al. 2001). Crank speed in handcycling around 50 rpm lead to increased mechanical efficiency (Kraaijenbrink et al. 2017). Further increasing velocity of propulsion can lead to reduced efficiency, increased joint accelerations and torque, and consequently lead to injuries (Mercer et al. 2006). Certainly, normal wheelchair propulsion has acceleration and deceleration phases and not only steady state speed as assumed in this optimization study. But here the chosen steady state speed is higher than the normal self-selected cadence, which is between 25 and 35 rpm (Rankin et al., 2012), and the authors believe that the selected 50 rpm steady state speed may lead to joint parameters equivalent to the short acceleration and deceleration phases experienced by

users during propulsion at lower cadences.

The dynamic optimization simulations were performed using the OpenSim-C++ API by accessing the OpenSim and Simbody libraries. The obtained simulation states files were further analyzed (e.g. muscle work and the normalized muscle force–length and force–velocity values) and processed in the OpenSim GUI and MS Excel.

## 3. Results

**Figure 2.**
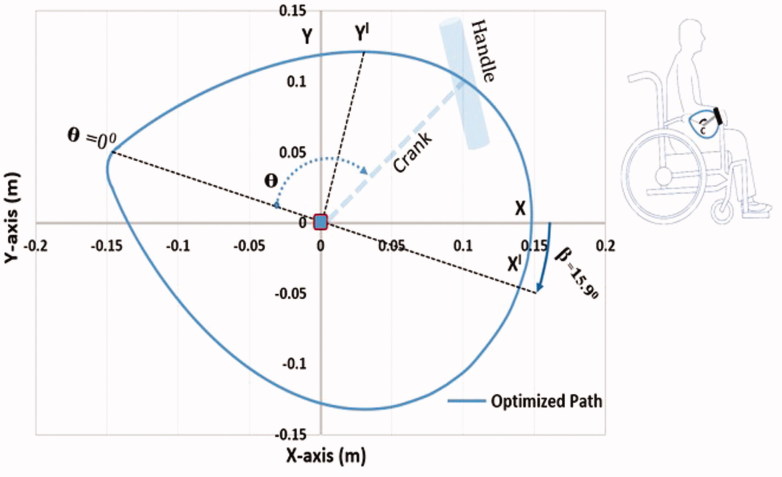
Dynamically optimized propulsion path with the centre C for HBP in the parasagittal plane defined by the wheels.

The dynamic optimization of the control variables at constant angular velocity of 50 rpm resulted in a shape as shown in Figure 2, with a circularity ratio (i.e. function of perimeter and area of the shape, a circle has a circularity of 1) of 0.951 and the optimized shape parameters *A* = 0.151 m, *B* = 0.152 m and *n* = 0.700. The propulsion path is tilted in clockwise direction (β = $15.950^{0}$) with respect to the ground frame. The optimization for the HBP resulted in a net propulsion power of 34.650 watts at 50 rpm.

**Figure 3.**
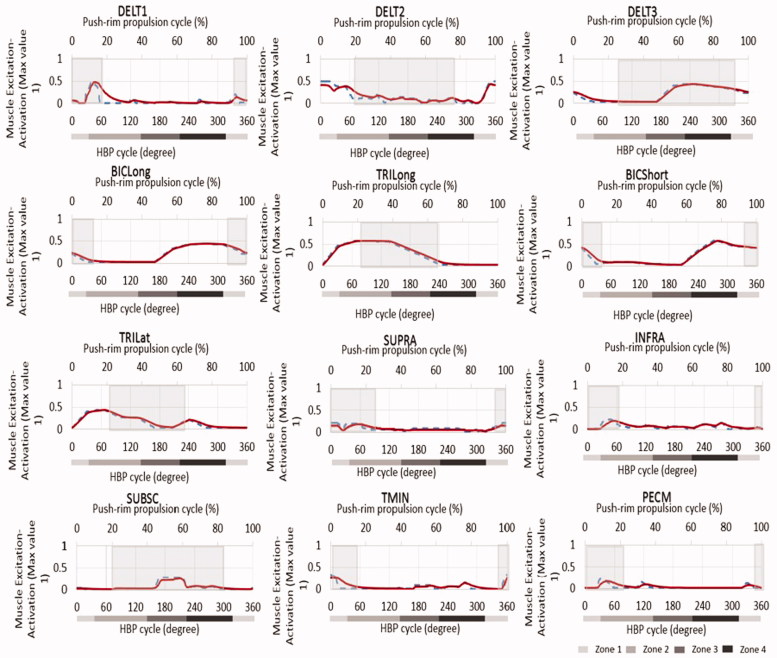
Optimized muscle activity patterns (only muscles for which a comparison to push rim propulsion is available), with the dark solid lines (muscle activations) and the dotted lines (muscle excitations) over one full propulsion cycle. The shaded regions indicate the phases in which the respective muscles were active during push-rim propulsion (Mulroy et al. 1996). The shaded bars below the diagrams show the propulsion zones.

**Figure 4.**
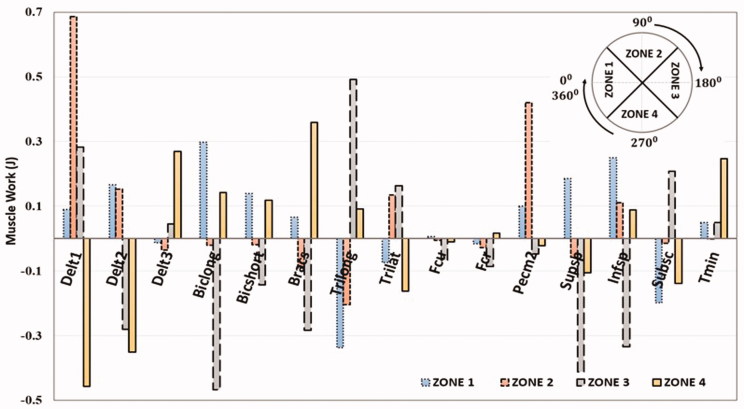
Net work done by the upper limb muscles (in joules) during the four zones of propulsion with the optimized handle path.

The optimized muscle excitation patterns from the simulation are shown in Figure 3. During push (zones 2, 3) mainly Delt1, Trilong, PecM, Infraspinatus, Teres Minor showed excitation, whereas during pull (zones 1, 4) mainly the muscles Delt3, BicShort, Biclong, Subscapularis were excited. Delt2 and Supraspinatus were active during parts of both, pull and push phases. Calculation of the net muscle work (in Joules) produced by the muscles during the four zones of propulsion (Figure 4) shows that Delt1, Delt3, PecM, Infraspinatus, Biclong, Bicshort, Brachialis, Trilong and Trilat contributed most to the net positive work during propulsion. The highest amount of positive work, 0.680 Joules, is produced by Delt1. Considerable amount of negative work was observed by Delt1 and BicLong in the regions of eccentric motion.

**Figure 5.**
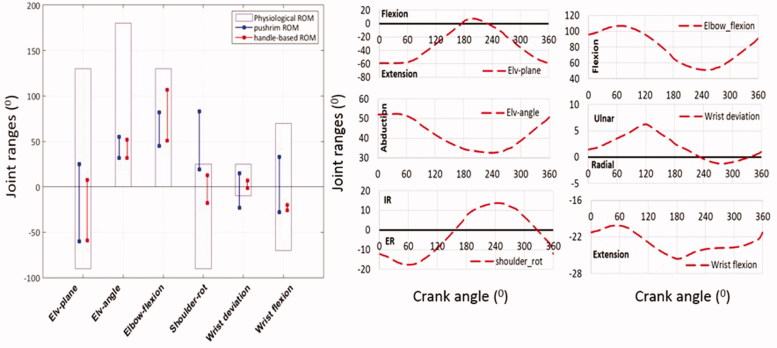
Comparison of the joint ranges of motion (ROM) of the upper extremity between push-rim propulsion and the HBP (handle-based propulsion) technique (Rankin et al. 2010; Morrow et al. 2014). Shoulder rotation Int/Ext(+/−).Wrist deviation Ulna/Radial(+/−) and wrist flexion Ext/Flex(−/+). The physiological range represents the anatomical joint range. The figures on the right side indicate the joint motion during HBP.

A comparison of the joint ranges of motion during standard push-rim propulsion and propulsion with the optimized shape shows that for the optimized shape, all joint ranges stay within their ergonomic regions, whereas during push-rim propulsion shoulder rotation and wrist deviation move outside the ergonomic ranges (Figure 5). Due to the fact that the joint motions from optimization were well within the ergonomic ranges and not at extreme limits, the effect of the coordinate restraining torques were not analysed explicitly as it will be minimal.

**Figure 6.**
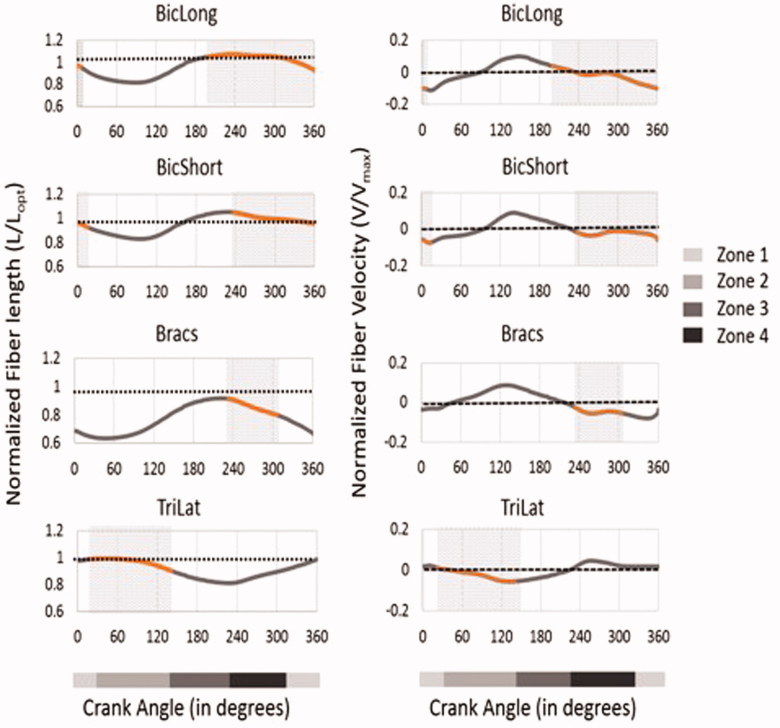
Normalized fiber length and normalized fiber shortening velocity of the muscles spanning the elbow joint during one full crank rotation. The dotted lines represent the optimal fiber length (L_opt_). In the normalized fiber velocity graph the muscle contraction is negative and muscle lengthening is positive. The maximum shortening velocity of each muscle was assumed to be 10 optimal fiber lengths per second (*V*_max_ = 10 L_opt_ s^−1^). The dark shaded areas in the graphs represent the regions with more that 20 percent of muscle activation.

**Figure 7.**
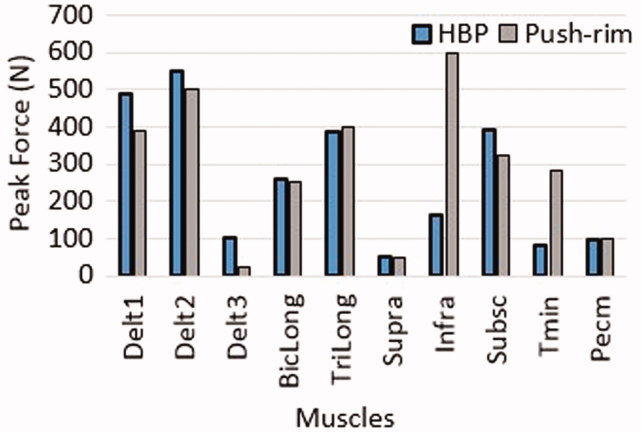
Peak muscle forces obtained from the computational simulation of HBP compared to dynamic optimization results for push rim propulsion (Morrow et al. 2014).

Figure 6 shows that all four muscles spanning the elbow joint were working close to their optimal fiber lengths, where they can generate highest active muscle forces, and with negative fiber velocity (contraction), meaning that they can generate positive muscle power, in the regions with activity above 20% (grey shaded regions). In addition, Figure 7 shows a comparison of the peak muscle forces during propulsion with HBP and push-rim at self-selected speeds (35 ± 8 rpm). Both propulsion modes produced near equal peak muscle forces, especially for the deltoid and the elbow muscle groups. Even though the comparison was performed to a lower cadence propulsion, the Infra and Tmin generated higher peak force when compared to HBP.

## Discussion

This study opens up for a new wheelchair propulsion movement, which is optimized for the musculoskeletal architecture of the upper extremity. The optimization at the chosen angular velocity of 50 rpm, resulted in a unique propulsion pattern for the HBP, having a circularity shape factor less than 1. This resembles the hand stroke pattern generated during wheelchair racing (Goosey et al. 2000) and a semi-circular pattern observed in the classic wheelchair stroke (Kwarciak et al. 2009). This pattern for the HBP is continuous, cyclic and improves hand contact during full propulsion cycle in contrary to push-rim propulsion. In addition, this dynamic movement pattern with alternate activation of agonist and antagonist muscles, increases dynamic muscle activity, which may increase blood circulation and help to postpone local muscle fatigue of the upper limb. Furthermore, the net propulsion power of 34.65 W generated from the HBP optimization supports the hypothesis that the HBP can produce sufficient power to propel a conventional wheelchair for daily life activities. This remains in agreement with previous studies that have indicated that a minimum of 30 W is required for a person to propel on a 3°–6° inclined slope, which demands higher muscular effort (Richter et al. 2007).

A comparison of the joint ranges of motion (ROM) between the stages of hand-rim propulsion (Rankin et al. 2010; Morrow et al. 2014) and HBP, clearly indicates that the HBP mechanism leads to motions which are in the ergonomic ranges for all joints, thus avoiding over-exertion of joints during the propulsion movement. In HBP, during the onset of the propulsion motion (pull phase) the shoulder is extended, abducted and externally rotated by the activation of muscles such as delt3, delt2, infraspinatus and supraspinatus. This motion subsequently leads to the push phase where the shoulder is flexed, adducted and internally rotated. The pattern of shoulder rotation is different to that observed in push-rim. The subscapularis in HBP has lower duration of activation when compared to push-rim and also facilitates greater contribution of the external rotators such as infraspinatus and teres minor, which may prevent the muscle imbalances leading to sub acromial impingement (Mulroy et al. 1996). Major joint excursions during wrist movements, which may cause CTS (Carpal Tunnel Syndrome) (Vanlandewijck,Veeger et al. 2001), are considerably reduced in HBP. The groups of muscles activated in pull and push zones were similar to wheelchair propulsion (Schantz et al. 1999).

The major elbow muscles, BicLong and TriLong exhibit large ranges of both positive and negative work during the propulsion zones in both HBP and push-rim propulsion (Rankin et al. 2012). In push-rim propulsion during the pull phase, BicLong – positive work and TriLong – negative work are observed and vice versa in push phase with BicLong absorbing force from the push-rim. In HBP, similar pattern of work done by BicLong and TriLong as in push-rim is noted (Rankin et al. 2011). The kinematics of the elbow joint moves from flexion to extension with the elbow flexor-extensor muscles shortening in regions, where the muscles are close to their optimal fiber length and velocity, which may result in increased muscle force production.

There are a few limitations in this study, which need to be addressed. First, the results were obtained in a simulation study using an experimentally validated 3D musculoskeletal model of a 50th percentile adult male but are not yet supported with experimental data. However, several studies have reported that the use of dynamic optimization techniques on 3D models closely resembled the experimental results (Pandy et al. 1992; Rankin and Neptune 2008; Morrow et al. 2014; Sharif Shourijeh and McPhee 2014; Saul et al. 2015). Second, the function of the trunk muscles has not been investigated in this study as the authors consider the HBP can be used over a larger population, not only persons with limited trunk function (SCI with higher lesion) but also disabled persons with intact trunk control (as e.g. leg amputees). Thirdly, the angular velocity of the crank was fixed to replicate an isokinetic propulsion since the constant velocity profile was needed to obtain a unique propulsion shape during the path optimization process. Assuming steady state propulsion at a low constant speed, straight forward over a leveled tiled surface the inertia of wheelchair and the related crank drive train dynamics were not explicitly modeled. As the objective function was designed to maximize the power for the optimal shape, the derived 34.65 W at handle is sufficient to overcome the minimum resistive forces experienced during wheelchair propulsion for the assumed conditions (Lin et al. 2015).Studies on cycling have reported that the crank inertial loads have minimum influence on the joint kinematics of users at a constant cadence of propulsion (Rankin and Neptune 2008). The effects of the rolling resistance and air resistance will be minimal (van der Woude,Veeger et al. 2001) on the assumed conditions and the addition of minor weights to the system has no effects on the joint kinematics (Bednarczyk and Sanderson 1995) for wheelchair propulsion.

This study offers some short and long term perspectives, a thorough experimental study is needed on the future developed HBP mechanism to test its functionality and efficiency on novice and veteran wheelchair users. There is also a wide scope in the industrial sector to develop a new wheelchair propulsion device for the disabled users.

## 4. Conclusion

This study describes the computational optimization of a novel handle based mechanism for wheelchair propulsion, which might be an interesting alternative to pushrim propulsion especially for long term wheelchair users, due to ergonomical joint angle ranges and lower muscle loads that might help to prevent injuries due to wheelchair propulsion.
